# Clinical implication of expression of vascular endothelial growth factor-C in metastatic lymph nodes of uterine cervical cancers

**DOI:** 10.1038/sj.bjc.6601963

**Published:** 2004-06-29

**Authors:** J Fujimoto, H Toyoki, E Sato, H Sakaguchi, T Tamaya

**Affiliations:** 1 Department of Obstetrics and Gynecology, Gifu University School of Medicine, 1-1 Yanagido, Gifu City 501-1193, Japan

**Keywords:** VEGF-C, metastatic lymph node, prognosis, uterine cervical cancer

## Abstract

Vascular endothelial cell growth factor (VEGF)-C levels were significantly (*P*<0.05) increased in 24 out of 40 metastatic lymph node lesions of uterine cervical cancers. The prognosis of the 24 patients with increased VEGF-C level in metastatic lymph node lesions was poor and the 24-month survival rate was 38%, while the rate of the 16 patients with no change in VEGF-C level in metastatic lymph node lesions was 81%. There was a significant (*P*<0.01) difference in the 24-month survival rates between the two groups. This is novel, direct evidence that VEGF-C might contribute to the aggressive lymphangitic metastasis in uterine cervical cancers, and that the increase in VEGF-C level from primary tumour to metastatic lymph node might be a prognostic indicator.

The presence of lymph node metastasis, recognised as the most common metastatic lesion, is critical to patient prognosis in uterine cervical cancers (Saigo *et al*, 1986; Noguchi *et al*, 1987, Robert *et al*, 1990; Chen *et al*, 1998). Vascular endothelial cell growth factor (VEGF)-C was cloned as a ligand of the Flt-4 (VEGFR-3) and KDR/flk-1 (VEGFR-2) receptor tyrosine kinases and recognised as a novel VEGF (Joukov *et al*, 1996; Lee *et al*, 1996). VEGF-C induces selective hyperplasia of the lymphatic vasculature, which is involved in the draining of interstitial fluid and in immune function, inflammation, and tumour metastasis (Jeltsch *et al*, 1997). Moreover, VEGF-C-induced lymphangiogenesis mediates tumour cell dissemination and the formation of lymph node metastases in transgenic mice with VEGF-C expression (Mandriota *et al*, 2001). The expression of VEGF-C in prostatic carcinoma cells has been implicated in lymph node metastasis (Tsurusaki *et al*, 1999). Gastric cancer cells producing VEGF-C induce the proliferation and dilation of lymphatic vessels, resulting in the invasion of cancer cells into the lymphatic vessels and lymph node metastasis (Yonemura *et al*, 1999), and the lymphatic invasion was significantly increased in VEGF-C-positive early gastric carcinoma (Kabashima *et al*, 2001). VEGF-C is involved in the progression of human gastric carcinoma, particularly via lymphangiogenesis, and VEGF-C expression at the invading edge of a gastric carcinoma is a sensitive marker for metastasis to the lymph nodes (Amioka *et al*, 2002). VEGF-C has an important role in lymph node metastasis of breast cancer even at its hormone-dependent early stage (Mattila *et al*, 2002). Proliferating lymphatics can occur in head and neck cancers and may in some cases contribute to lymph node metastasis (Beasley *et al*, 2002). The involvement of VEGF-C expression in the promotion of lymph node metastasis in cervical cancer has been demonstrated. Furthermore, examination of VEGF-C expression in biopsy specimens may be beneficial in the prediction of pelvic lymph node metastasis (Hashimoto *et al*, 2001).

Although lymph node metastasis has been analysed by the manner of VEGF-C expression in primary tumours, the VEGF-C expression status in metastatic lymph nodes as a result has not been directly investigated. This status prompted us to investigate the clinical significance of VEGF-C expressed in lymph nodal metastasis of uterine cervical cancers.

## MATERIALS AND METHODS

### Patients

**Figure 1:**
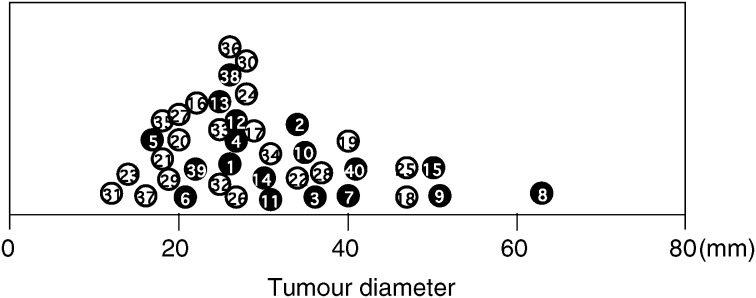
Tumour diameter of all cases. Alive and deceased cases are numbered in ○ and •, respectively.

Consent for the following studies was obtained from all patients and the Research Committee for Human Subjects, Gifu University School of Medicine. A total of 40 patients ranging from 39 to 82 years of age underwent curative resection for cervical cancer stage IIb. All cases involved direct extension to the parametrium histopathologically, and had lymph node metastasis. The tumour diameters are shown in Figure 1

. The therapy after operation was whole pelvic irradiation covering the lymph node areas for all cases. Their 24-month survival rates were assessed at the Department of Obstetrics and Gynecology, Gifu University School of Medicine, between January 2000 and March 2002. None of the patients had received any preoperative therapy. A part of each tissue of uterine cervical cancers was obtained immediately after hysterectomy and was snap-frozen in liquid nitrogen to determine the levels of VEGF-C, and a neighbouring part of the tissues was submitted for histopathological study. Perfect lymphadenectomy was performed with ease, and all lymph nodes taken were divided into two; one piece was used for histopathological examination and immunohistochemical staining, and the other was used for enzyme immunoassay. The clinical stage of uterine cervical cancers was determined by International Federation of Obstetrics and Gynecology (FIGO) classification (FIGO News, 1989).

### Immunohistochemistry

Sections (4 *μ*m) of formalin-fixed paraffin-embedded tissues were cut with a microtome and dried overnight at 37°C on a silanised-slide (Dako, Carpinteria, USA). Samples were deparaffinised in xylene at room temperature for 80 min and washed with a graded ethanol/water mixture and then with distilled water. The samples for VEGF-C antigen were soaked in citrate buffer (10 mM citrate), microwaved for 10 min, and then treated with 3% H_2_O_2_ in a phosphate buffer (PBS) at room temperature for 20 min. The protocol for a DAKO LSAB2 Kit, Peroxidase (Dako) was followed for each sample except for the incubation condition for the first antibody. Goat anti-human VEGF-C (200 *μ*g ml^−1^, Santa Cruz Biotechnology, Santa Cruz, CA, USA) was used at a dilution of 1 : 50 as the first antibody with incubation at 4°C overnight. The addition of the first antibody, goat anti-human VEGF-C, was omitted in the protocols for negative controls of VEGF-C.

The results of immunohistochemical staining for VEGF-C were semiquantitatively evaluated as described by McCarty *et al* (1985). The evaluations were recorded as the percentage of positive-stained cells in each of five intensity categories that were denoted as 0, 1, 2, 3, 4, and 5. Each stained section was given a histochemical score (histoscore, HS) calculated by the formula: Σ (*i*+1) × Pi, in which *i*=cellular staining intensity (range 1–4, 0 indicates no staining) and Pi=percentage of stained cells.

### Enzyme immunoassay for determination of human VEGF-C antigen

All steps were carried out at 4°C. Tissues (wet weight, 10–20 mg) were homogenised in HG buffer (5 mM Tris-HCl, pH 7.4, 5 mM NaCl, 1 mM CaCl_2_, 2 mM ethyleneglycol-bis-[*β*-aminoethyl ether]-*N*,*N*,*N*′,*N′*-tetraacetic acid, 1 mM MgCl_2_, 2 mM dithiothreitol, 25 *μ*g ml^−1^ aprotinin, and 25 *μ*g ml^−1^ leupeptin) with a Polytron homogeniser (Kinematics, Luzern, Switzerland). This suspension was centrifuged in a microfuge at 10 000 **g** for 3 min to obtain the supernatant. The protein concentration of samples was measured by the method of Bradford (1976) to standardise VEGF-C antigen levels.

VEGF-C levels in the samples were determined using a VEGF-C ELISA kit (MBL, Nagoya, Japan). The levels of VEGF-C were standardised with the corresponding cellular protein concentrations.

### Statistics

The 24-month survival rate was calculated according to the Kaplan–Meier method and analysed by the log-rank test. VEGF-C histoscores and levels were measured from three parts of the same tissue in triplicate. Statistical analysis was performed with a paired *t*-test. Differences were considered significant when *P*-value was less than 0.05.

## RESULTS

**Figure 2:**
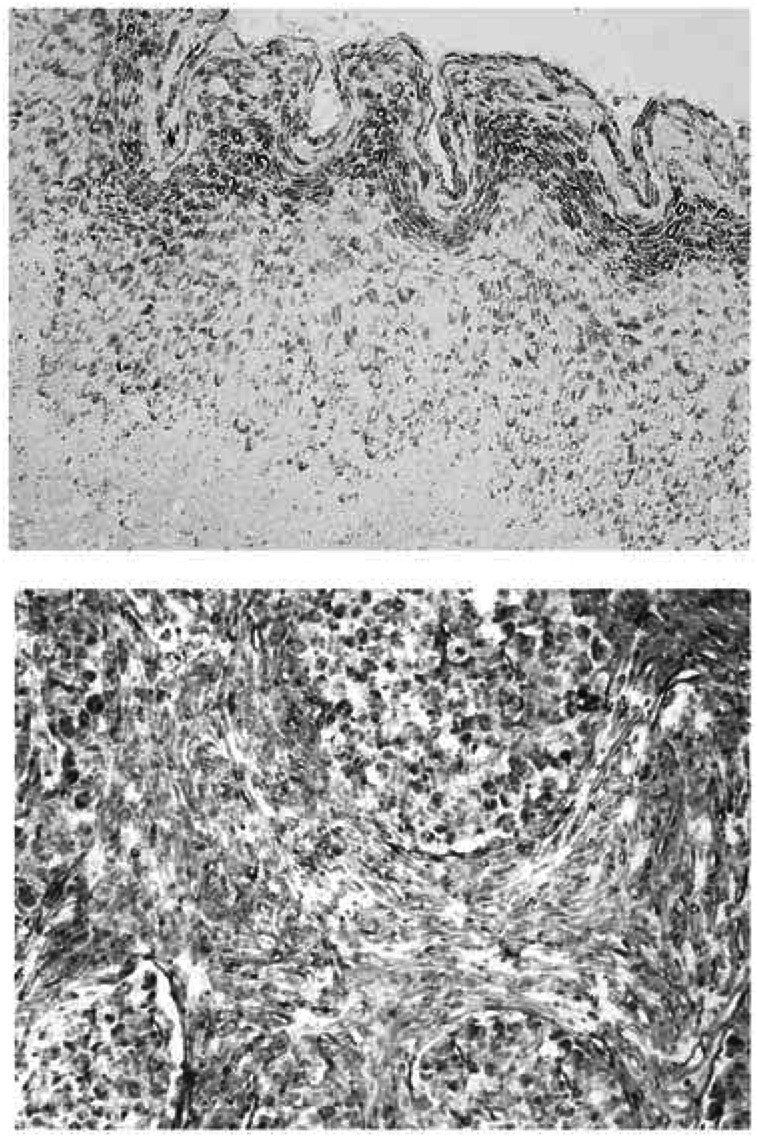
Immunohistochemical staining for VEGF-C in primary tumour and metastatic lymph node lesion in a representative case of uterine cervical cancer (original magnification × 200). A case of large cell nonkeratinising squamous cell carcinoma of the uterine cervix, metastatic lesion in cardinal lymph nodes. The addition of the first antibody, goat anti-human VEGF-C antibody, was omitted in the protocols for negative controls of VEGF-C.

Immunohistochemical staining for VEGF-C in a representative case of large cell nonkeratinising squamous cell carcinoma is shown in Figure 2

**Figure 3:**
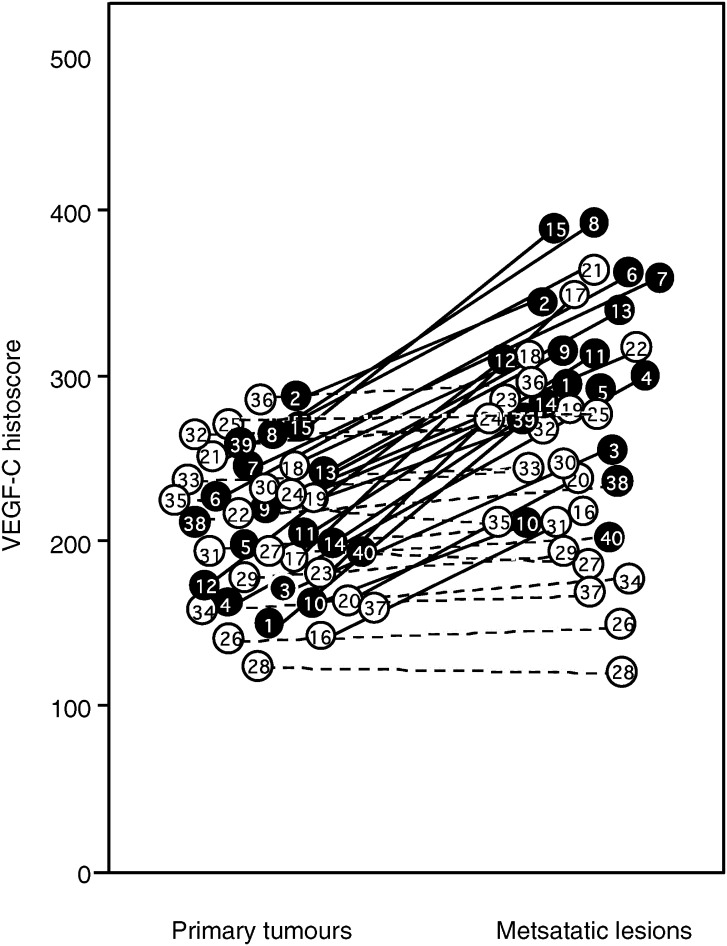
VEGF-C histoscores in primary tumour and in metastatic lymph node lesion of uterine cervical cancers. VEGF-C histoscores were determined as described in Materials and methods. In the primary tumours and corresponding metastatic lymph node lesions, alive and deceased cases are numbered in ○ and •, respectively. Bold lines, increased significantly (*P*< 0.05 *vs* each primary tumour) from the primary tumour to the corresponding metastatic lymph node lesion. Broken lines, no significant change between the primary tumour and the corresponding metastatic lymph node lesion.

. VEGF-C was dominantly distributed in the cancer cells. As shown in Figure 3

, VEGF-C histoscores in 24 out of 40 metastatic lymph node lesions of uterine cervical cancers (shown with bold lines) were significantly (*P*<0.05) higher than in the corresponding primary tumours, while the scores in the other 16 lesions (shown with broken lines) were not significantly altered.

**Figure 4:**
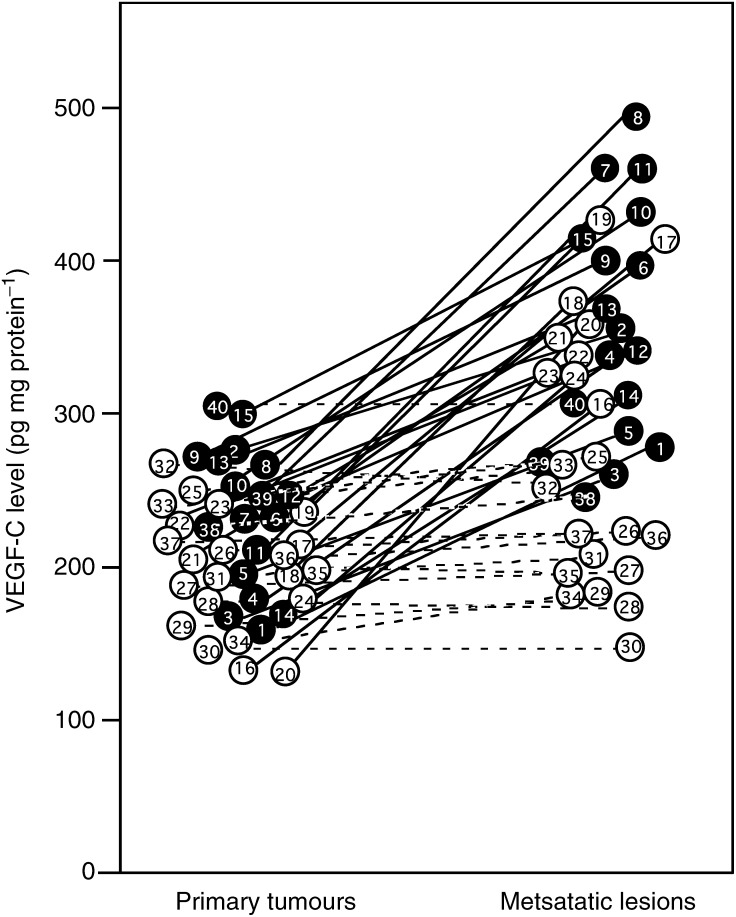
VEGF-C levels in primary tumour and metastatic lymph node lesions of uterine cervical cancers. VEGF-C levels were determined using a VEGF-C ELISA kit (MBL, Nagoya, Japan). Each level is the mean±s.d. of nine determinations from three parts of the same tissue in triplicate. In the primary tumours and the corresponding metastatic lymph node lesions, alive and deceased cases are numbered in ○ and •, respectively. Bold lines, increased significantly (*P*<0.05 *vs* each primary tumour) from the primary tumour to the corresponding metastatic lymph node lesion. Broken lines, no change between the primary tumour and the corresponding metastatic lymph node lesion.

**Figure 5:**
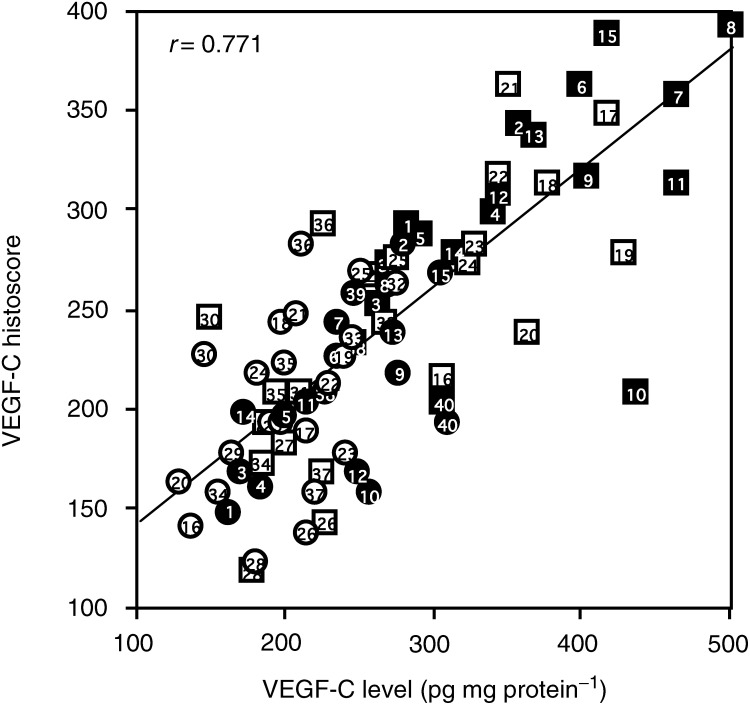
Correlation between VEGF-C levels and VEGF-C histoscores in primary tumour and metastatic lymph node lesions of uterine cervical cancers. Alive and deceased cases in the primary tumours are numbered in ○ and •, respectively. Alive and deceased cases in the metastatic lymph nodes are numbered in □ and ▪, respectively.

The VEGF-C level in the primary tumours was less than approximately 300 pg mg^−1^ protein (Figure 4

). On the other hand, the VEGF-C level in 24 out of 40 metastatic lymph node lesions of uterine cervical cancers (shown with bold lines) was remarkably (*P*<0.05) higher than in the corresponding primary tumours, while the level in the other 16 lesions (shown with broken lines) was not significantly altered. The cases with increased VEGF-C as identified by immunohistochemical staining were the same cases as those identified by the ELISA (Figures 3 and 4). The VEGF-C levels significantly (*P*<0.001) correlated with the VEGF-C histoscores as shown in Figure 5

.

**Figure 6:**
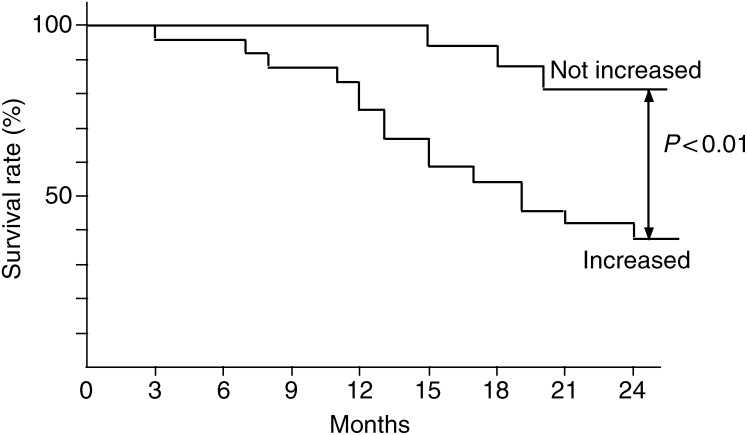
Survival rate after curative resection for uterine cervical cancers. Patient prognosis was analysed with a 24-month survival rate. Increased, the cases with significantly increased VEGF-C level from the primary tumour to the metastatic lymph node lesion; not increased, the cases with no change in VEGF-C level from the primary tumour to the metastatic lymph node lesion.

The prognosis of the 24 patients with increased VEGF-C level in the metastatic lesions was poor and the 24-month survival rate was 38% (9 of 24), while the rate of the other 16 patients with unchanged VEGF-C level in metastatic lesions was 81% (13 of 16). There was a significant (*P*<0.01) difference in the 24-month survival rates between the two groups (Figure 6

).

## DISCUSSION

In the present study, the VEGF-C level in 24 out of 40 metastatic lymph node lesions of uterine cervical cancers was remarkably increased, and the prognosis of the 24 patients was poor. Many authors have reported that VEGF-C contributes to lymphogenous metastasis. If indeed it does, a cancer cell enriched with VEGF-C in the cancer tissue may selectively work on lymphogenous metastasis, and the expression of VEGF-C would be increased from the primary tumour to the corresponding metastatic lymph node lesions. Furthermore, such a phenomenon would be induced in a cascade manner, causing VEGF-C expression to be increased in the secondary metastatic lymph node lesions, resulting in poor clinical prognosis in such cases. On the other hand, in the cases with no change in the VEGF-C level, VEGF-C might not mainly or directly contribute to lymph node metastasis. Although lymph node metastasis appears to be regulated by additional factors besides VEGF-C, such a cascade of lymph node metastasis might be less active in these cases, resulting in comparatively better patient prognosis. This is novel, direct evidence that VEGF-C might contribute to aggressive lymphangitic metastasis, and that the increase in VEGF-C level from primary tumour to metastatic lymph nodes might be a prognostic indicator. Therefore, VEGF-C might be a key signal to evoke and maintain the cascade of lymph node metastasis, and may be useful as a tumour marker for patient prognosis and a molecular target for treatment.
